# The diagnostic value of hysterosalpingo-contrast sonography in female infertility of the fallopian tube

**DOI:** 10.12669/pjms.39.4.7462

**Published:** 2023

**Authors:** Chunying Li, Yan Huang, Li Xie, Xin Huang

**Affiliations:** 1 Chunying Li, Department of Ultrasound, Beijing University of Chinese Medicine Shenzhen Hospital, Shenzhen, 518172, P.R. China; 2 Yan Huang, Department of Ultrasound, Longgang District People Hospital of Shenzhen, Shenzhen, 518172, P.R. China; 3 Li Xie, Department of Ultrasound, Beijing University of Chinese Medicine Shenzhen Hospital, Shenzhen, 518172, P.R. China; 4 Xin Huang, Department of Ultrasound, Shenzhen Longgang District Maternity & Child Health Care Hospital, Shenzhen, 518172, P.R. China

**Keywords:** Fallopian tube, Female infertility, Hysterosalpingo-contrast-sonograghy, Laparoscopy

## Abstract

**Objective:**

To explore the clinical diagnostic value of hysterosalpingo-contrast sonograghy (HyCoSy) in female infertility of the fallopian tube.

**Methods:**

One hundred nineteen female infertility patients who underwent laparoscopy in Shenzhen Hospital of Beijing University of Chinese medicine (Longgang) and Longgang District People’s Hospital of Shenzhen City from June 2019 to December 2021. Patients diagnosed with fallopian tube obstruction; 119 patients included 233 fallopian tubes (five patients had the affected fallopian tubes removed due to ectopic pregnancy) were selected for HyCoSy, and then the results of laparoscopic examination were taken as the gold standard for diagnosis. The authenticity of the diagnostic test was evaluated using four grid table data, and the consistency of the two diagnostic methods of hysterosalpingography and laparoscopy was compared by Kappa test.

**Results:**

Of the 233 fallopian tubes assessed, 139 were unobstructed, 50 were blocked at the proximal end and 44 were blocked at the distal end by laparoscopy. The results of HyCoSy showed that 115 were unobstructed, seventy three was considered proximal obstruction and 45 were distal obstruction. When compared to laparoscopy, the accuracy of HyCoSy in the diagnosis of tubal patency, proximal obstruction and distal obstruction was 74.2%, 78.1% and 80.7%, respectively. Two methods had good consistency in the diagnosis of tubal patency (Kappa=0.486) and proximal tubal obstruction (Kappa=0.444), and poor consistency in the diagnosis of distal tubal obstruction (Kappa=0.375).

**Conclusion:**

Laparoscopy and HyCoSy are both useful in the diagnosis and etiological analysis of female infertility. HyCoSy can be the first choice, those who have doubts about the screening results can actively carry out laparoscopy to further improve the accuracy of diagnosis and etiological analysis of this disease.

## INTRODUCTION

The fallopian tube is the place where eggs are fertilized and are divided. Fertilization is promoted through peristalsis, cilia swing and oviduct fluid flow, and the fertilized eggs are sent to the uterine cavity.^1^ When the structure and function of the fallopian tube are abnormal, the functions of collecting, fertilizing and transporting fertilized eggs decline, leading to infertility.^2^ The incidence of female infertility in China is ~7.0%-10.0%, which seems to be increasing in recent years. Of this incidence 25.0%-35.0% cases of female infertility are diagnosed as tubal factor infertility.^3^ Choosing an effective method of diagnosis and the underlying cause of infertility is important to provide individualized pregnancy assistance.^4^

Typically, the clinical diagnosis of female infertility starts with the evaluation of fallopian tube patency, through three methods, HyCoSy, laparoscopy, and salpingoscopy. HyCoSy is considered a first-line screening method, is a relatively simple operation causing limited trauma. The ultrasonic imaging agent is sulfur hexafluoride, which has a high viscosity, and insufficient pressure or dosing of the contrast agent can cause fallopian tube spasm or a false positive.^5^ Using laparoscopy, the fallopian tube and its surrounding tissues can be observed, and the cause of infertility can be determined. Currently, laparoscopy is the “gold standard” for the diagnosis of fallopian tube patency in clinic. However, laparoscopy is associated with risk of intestinal perforation and bleeding, can be traumatic and relatively expensive, so it cannot be widely used.^6^ The purpose of this study was to examine the clinical diagnostic value of both laparoscopy and HyCoSy in the diagnosis and analysis of female infertility, and to provide some basis for the treatment of female infertility.

## METHODS

From June 2019 to December 2021, 119 female patients with infertility who underwent laparoscopy in Shenzhen Longgang District Hospital of traditional Chinese medicine and Shenzhen Longgang District People’s hospital with oviduct obstruction were selected for HyCoSy examination, and then the results of laparoscopic examination were taken as the gold standard for diagnosis. The authenticity of the diagnostic test was evaluated using four grid table data, and the consistency of the two diagnostic methods of hysterosalpingography and laparoscopy was compared by Kappa test All procedures performed in study involving human participants were in accordance with the ethical standards of the institutional and/or national research committee(s) and with the Helsinki Declaration (as revised in 2013). Written informed consent was obtained from the patient or legal guardian. The medical ethics Committee of our hospital approved this study (No. SZLDH2022LSYM-127, Date: 2022-08-05).

### Inclusion criteria:

- Women who have regular sex with the same sexual partner for more than one year and fail to conceive without contraception.
- Voluntarily accept laparoscopy and ultrasound-guided HyCoSy.
- The interval between HyCoSy and laparoscopy is less than one year.
- Age ≤50 years old.

### Exclusion criteria:

- Contraindications of laparoscopy and HyCoSy examination.
- Congenital abnormality of reproductive tract.
- Ovulation function, immune factors and male semen abnormalities.
- Bilateral salpingectomy.
- Patients with severe primary diseases, organ dysfunction and malignant tumors.
- Cognitive, mental and language communication disorders.
- Incomplete medical records.

**Fig.1:**
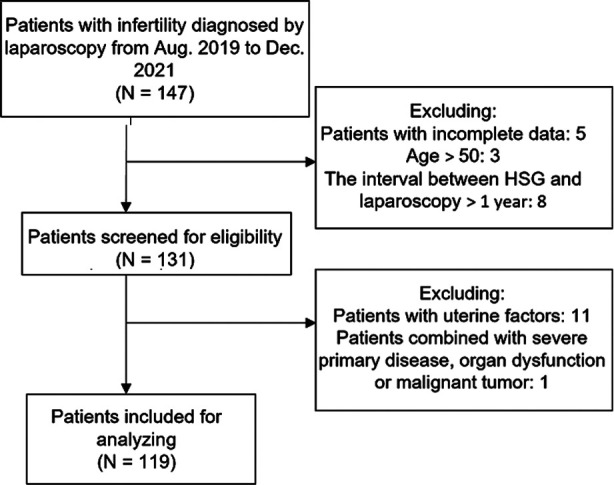
Patient screening flowchart.

A flow chart of patient screening can be seen in Fig.1. Laparoscopy was carried out 3~7 days after menstruation, and TV laparoscopy and instruments were selected. After general anesthesia, the subject was positioned into the bladder lithotomy position. The abdominal operation field was disinfected as was the vulva and vagina. Sterile towels were put down and a uterine lifter was placed (30°~40° head high and feet low). A 1cm incision was made on the umbilical margin and the pneumoperitoneum needle was punctured into the abdominal cavity. CO_2_ gas (2.6L) was filled to form an artificial pneumoperitoneum and 10mmTrocar was inserted. The second and third puncture points were made on the lower abdomen and 5mm trocar was inserted. An artificial pneumoperitoneum was established with 13mmHg air pressure after confirming that the abdominal organs were not damaged. A 10mm trocar was inserted followed by the microscope which allowed for abdominal exploration and observation of the patient’s fallopian tube and its accessories. A double chamber air bag was inserted into the uterine cavity through the vagina, 3ml of 0.9% sodium chloride was injected to form a water bag to fill the uterine cavity, while 20ml of methylene blue was injected perfusing the fallopian tube and allowing observation of fallopian tube patency. The same doctor carried out all film reading and diagnosis, and the classification of diagnostic results included.^7^,^8^

### Unobstructed:

No abnormal shape of fallopian tube and surrounding adhesion observed, and methylene blue can be seen flowing smoothly through the umbrella end.

### Proximal obstruction:

The fallopian tube is not developed, the operator feels increased resistance, the uterine horn bulges, and no methylene blue outflow is found at the umbrella end.

### Distal obstruction:

The umbrella end of the fallopian tube is clearly expanded and blue, and no methylene blue flows out of the umbrella end.

HyCoSy was carried out 3-7 days after menstruation. The patient was instructed to empty their bladder and bowels, flush their vagina, and lie on the examination bed in the bladder lithotomy position. Disinfection of the observation area was completed and the vagina and cervix were exposed using a vaginal speculum and disinfected with iodine. A disposable double lumen balloon angiography catheter was slowly inserted into the uterine cavity through the cervix, and 1.5~3ml of saline was injected and the catheter was fixed. An ultrasonic diagnostic instrument (E10, GE of the United States) was used to conduct three-dimensional scanning to measure the volume of bilateral fallopian tube contrast. The contrast agent was configured and four-dimensional ultrasonic scanning was completed. The contrast agent was pushed at a constant speed and dynamic contrast was used to image for multi-angle observation. After completing the examination, the same doctor carried out all film reading and diagnosis. The classification of diagnosis results included.^9^,^10^

**Fig.2:**
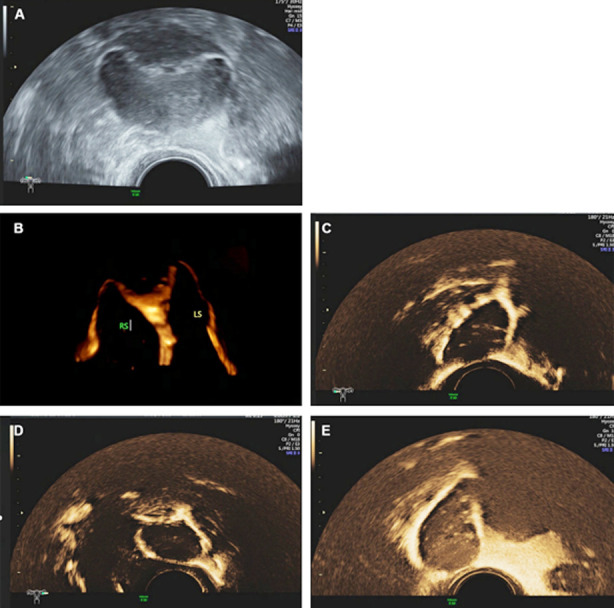
2A. Overflow of contrast medium in bilateral uterine horn and development of proximal fallopian tube. 2B. Whole course imaging of bilateral fallopian tubes. 2C. The contrast medium around the right ovary is strongly echoic and circularly diffused. 2D. The contrast medium around the left ovary is strongly echoic and circularly diffused. 2E. Bilateral umbrella end overflow and pelvic diffusion.

**Fig.3:**
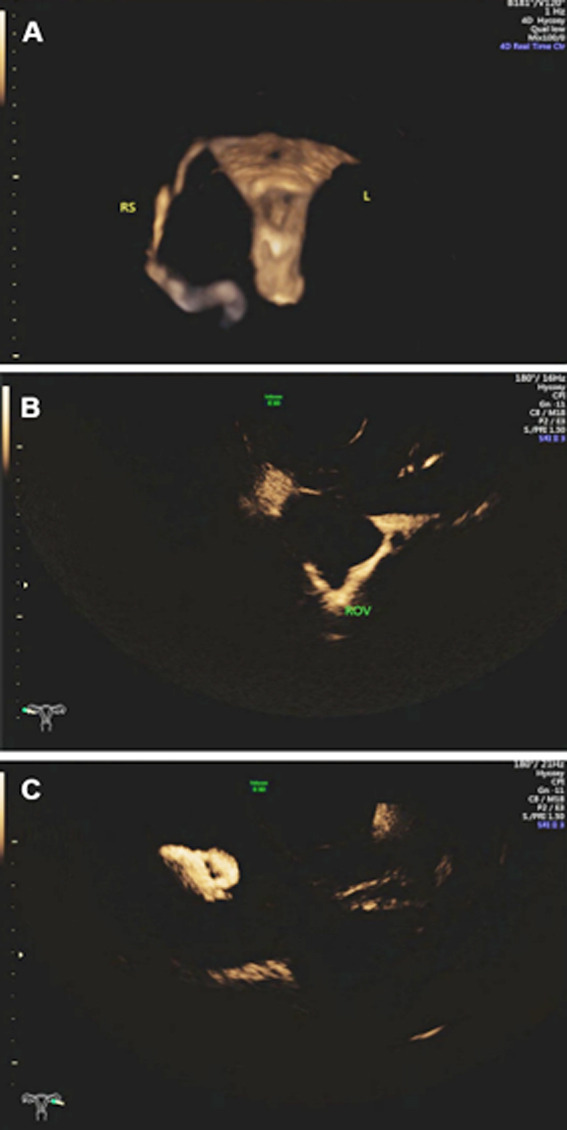
3A. The right fallopian tube is developed while the left fallopian tube is not developed. 3B. Diffuse contrast media can be seen in the right ovary. 3C. No contrast diffusion is found in the left ovary.

**Fig.4:**
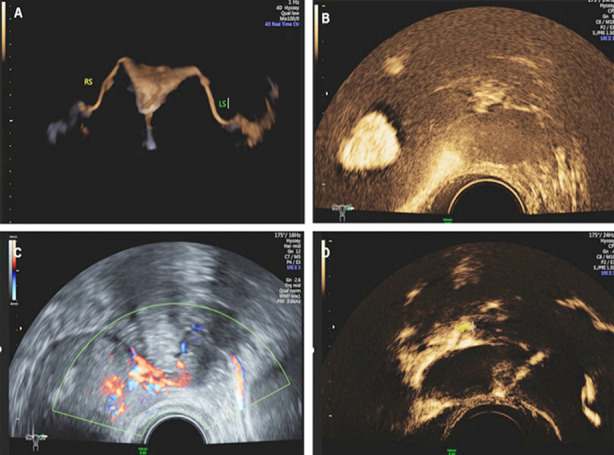
4A. Bilateral fallopian tube development. 4B. No dispersion is found in the left ovary. 4C. No contrast agent overflow is found at the left umbrella end. 4D. Diffusion and contrast agent overflow can be seen in the right ovary.

1) Unobstructed. The morphology of the uterine cavity of the uterus and fallopian tubes is in a normal state, and contrast media diffused from the umbrella ends of both fallopian tubes can be seen in the pelvic cavity (Fig.2A-2E).
2) Proximal obstruction. No fallopian tube development is visible, only contrast agent dispersion can be seen at the uterine horn. (Fig.3A-3C).
3) Distal obstruction. Fallopian tube development can be seen in the whole process, and cystic expansion can be seen, with a small amount of or no contrast medium diffusion in the pelvic cavity (Fig.4A-4D).

### Evaluation of diagnostic results:

Detection of tubal patency, proximal tubal obstruction and distal tubal obstruction by the two methods compared. The diagnostic results were judged by accuracy, specificity and sensitivity. The calculation formula: true positive is represented by a, false positive by b, false negative by c, true negative by d, accuracy = (a+d) / total number of cases, specificity =d/ (b+d), sensitivity =a/ (a+c), positive predictive value = a/ (a+b), negative predictive value = d/ (c+d).

### Statistical analysis:

Spss22.0 statistical software was used for data analysis. The diagnostic data was expressed in n (%), an *χ^2^* test was performed. When *p*<0.05, the difference was considered statistically significant. Kappa coefficient was used to test the consistency between the two methods.

## RESULTS

A total of 119 patients, with 47 cases of primary infertility and 72 cases of secondary infertility were included in this study. The patients ranged in age from 22~50 years, with an average of 32.1 ± 5.0 years. The duration of infertility ranged from 1-6 years, with an average of 3.5 ± 1.2 years. Of the 119 patients, five had ectopic pregnancies and the affected side of the fallopian tubes were removed. One hundred ninteen patients included 233 fallopian tubes (five patients had the affected fallopian tubes removed due to ectopic pregnancy) were analyzed. As shown in Table-I, the accuracy of HyCoSy in the diagnosis of fallopian tube patency, proximal obstruction and distal obstruction was 74.2%, 78.1% and 80.7%, respectively. Further calculation of sensitivity and specificity showed that the sensitivity and specificity of fallopian tube patency diagnosis were 69.8% and 80.9%. The sensitivity and specificity of proximal occlusion were 72.0% and 79.8%, respectively. The sensitivity of HyCoSy was 50.0%, and the specificity was 87.8%. The diagnostic consistency of the two methods was good when diagnosing fallopian tube patency, proximal obstruction and distal obstruction (Table-II).

**Table-I:** Diagnostic sensitivity and specificity of HyCoSy (%).

Oviduct	Sensitivity	Specificity	Positive predictive value	Negative predictive value	Diagnostic accuracy
Unobstructed fallopian tube	52.2%	88.6%	75.0%	73.8%	74.1%
Proximal occlusion	78.4%	79.7%	64.4%	88.7%	79.3%
Distal occlusion	69.7%	83.1%	62.2%	87.3%	79.3%

**Table-II:** The diagnostic consistency of the two methods.

Oviduct	Laparoscopy	HyCoSy	p-Value	Kappa	Consistency
Unobstructed fallopian tube	46	32	<0.001	0.430	moderate agreement
Proximal occlusion	37	45	<0.001	0.550	moderate agreement
Distal occlusion	33	37	<0.001	0.510	moderate agreement

## DISCUSSION

This study explored the clinical diagnostic value of HyCoSy in female infertility. HyCoSy is considered a first-line method for diagnosis of infertility, and is non-invasive, low cost and provides effective observation of fallopian tube morphology with a clinical coincidence rate of diagnosis is about 50.0%-90.0%.^11^ Our results show the accuracy of HyCoSy in the diagnosis of tubal patency to be 74.2%, with a good consistency and a sensitivity of 69.8%. HyCoSy resulted in a specificity of 80.9%, a positive predictive value of 49.3%, and a negative predictive value of 91.3%. Pande B et al^12^ showed that hysterosalpingography could save about 30.0% of female infertility patients from laparoscopy. The sensitivity and negative predictive values presented here are lower than those reported by Gundu Z^11^ and Chowdhury.^13^ However, their accuracy, specificity and positive predictive value are higher than those reported by Gundu Z and Chowdhury, but their specificity and negative predictive value are similar to those reported by Foroozanfard.^11^,^13^,^14^

Laparoscopy is the gold standard for the diagnosis of tubal diseases, and clearly shows the morphology of fallopian tubes, comprehensively observes the peristalsis of fallopian tubes, pelvic cavity and other conditions. Both laparoscopy and HyCoSy have good consistency in the diagnosis of proximal and distal fallopian tube obstruction, but the specificity and sensitivity are not ideal. In the diagnosis of proximal tubal obstruction, the accuracy of HyCoSy is 78.1%, the sensitivity is 72.0%, the specificity is 79.8%, the positive predictive value is 49.3%, and the negative predictive value is 91.3%, which is inconsistent with the research of Ngowa et al,^15^ suggesting that HyCoSy has a certain false positive rate in the tubal patency test. It is possible that either a tubal or oviduct spasm may occur under the influence of pain, stimulation, tension and other factors in the examination process affecting the diagnostic results.^16^,^17^ It is also possible for endometrial polyps to block the opening of the fallopian tubes, or that HyCoSy may have the effect of dredging the fallopian tubes during the examination, which may make the opposite results appear during laparoscopy.^18^ In the diagnosis of distal tubal obstruction, the sensitivity, specificity, positive predictive value and negative predictive value of ultrasound-guided HyCoSy are 50.0%, 87.8%, 48.9% and 88.3%, the sensitivity and positive predictive value are far lower than those of Ngowa et al,^15^ suggesting that if HyCoSy shows hydrosalpinx, it is extremely unlikely to show no hydrosalpinx again by laparoscopy. Lin Y H et al^19^ research suggests that for such patients, regardless of whether the hydrosalpinx is serious, laparoscopic salpingoplasty should be selected in time for treatment. In addition, as contrast agent can affect thyroid function, HyCoSy should be used with caution in people with thyroid disease.^20^

### Limitation of the study:

It includes small sample size. HyCoSy interpretation has certain subjectivity, which depends on the experience and technical level of the film reader, and may make the diagnostic results one-sided. The time interval between HyCoSy and laparoscopy may affect the difference of diagnostic results between the two methods.

## CONCLUSION

Laparoscopy and HyCoSy have a positive relationship in the diagnosis and etiological analysis of female infertility. HyCoSy should be the first choice for routine screening in clinic. Those who have doubts about the screening results can actively carry out laparoscopy to further improve the accuracy of diagnosis and etiological analysis of this disease.

### Authors’ contributions:

**CL and YH:** Conceived and designed the study.

**LX and XH:** Collected the data and performed the analysis.

**CL and YH:** Were involved in the writing of the manuscript and is responsible for the integrity of the study.

All authors have read and approved the final manuscript.
